# Approximating vaccine delivery costs to reach zero-dose children: a Bayesian meta-regression analysis

**DOI:** 10.1093/heapol/czag085

**Published:** 2026-07-02

**Authors:** Allison Portnoy, Emma Clarke-Deelder, Taylor A Holroyd, Daniel R Hogan, Tewodaj Mengistu

**Affiliations:** Department of Global Health, Boston University School of Public Health, 801 Massachusetts Avenue, Boston, MA 02118, United States; Center for Health Decision Science, Harvard T.H. Chan School of Public Health, Boston, MA 02115, United States; Department of Epidemiology and Public Health, Swiss Tropical & Public Health Institute, Kreuzstrasse 2, Allschwil 4123, Switzerland; Gavi, the Vaccine Alliance, Chem. du Pommier 40, Le Grand-Saconnex 1218, Switzerland; Gavi, the Vaccine Alliance, Chem. du Pommier 40, Le Grand-Saconnex 1218, Switzerland; Gavi, the Vaccine Alliance, Chem. du Pommier 40, Le Grand-Saconnex 1218, Switzerland

**Keywords:** vaccines, costs, decision-making

## Abstract

The Immunization Agenda 2030 calls for reaching all people with immunization services, including “zero-dose” children—children who have not received any routine vaccines. To plan and finance efforts to fully vaccinate these children and improve coverage and equity, decision-makers need reliable cost estimates. However, primary data on the costs of reaching zero-dose children, typically part of disadvantaged and hard-to-reach populations, are scarce. This study approximates these costs using standardized, country-level estimates of vaccine delivery unit costs for outreach delivery in low- and middle-income countries (LMICs). We extracted outreach delivery cost per dose estimates for childhood immunization services from the 2024 update of the Immunization Delivery Cost Catalogue. Using these data, we developed a meta-regression model to estimate standardized outreach vaccine delivery unit costs. The generalized linear model assumed a Gamma-distributed outcome with a log link and included both country-level and study-level predictors: study year, economic or financial cost basis, routine or campaign delivery, and full or incremental costing approach. The fitted model was used to estimate 2024 outreach delivery costs per dose for 129 LMICs. The model was estimated using 48 observations from 19 countries focused on outreach or mobile vaccine delivery. The best-fitting specification included diphtheria-tetanus-pertussis coverage, per capita gross domestic product, and under-five population size as predictors. For 2024, the predicted mean economic cost per dose was $8.65 (95% credible interval $2.33–23.71), averaged across all 129 LMICs. To fully immunize a zero-dose child with 13 recommended vaccinations, the equivalent cost estimate was $112.45 ($30.29–308.23). Reaching zero-dose children is crucial for improving equity in global health, and estimates of the costs of doing so are needed to inform budgeting for immunization programs. These meta-regression-based cost estimates can help countries to improve budgeting, planning, and resource allocation for efforts to reach zero-dose children.

Key messages

- Despite global commitments under the Immunization Agenda 2030, millions of ‘zero-dose’ children in low- and middle-income countries (LMICs) remain unvaccinated, often due to poverty, geographic inaccessibility, or conflict.
- Given the resource intensity of collecting data on the costs of reaching zero-dose children, existing evidence is primarily available for a small number of countries from often small-scale implementation studies, providing important context-specific insights while highlighting the need for broader standardized estimates.
- Using a Bayesian meta-regression framework, this study estimated standardized, country-level estimates of vaccine delivery costs based on data for outreach delivery—a proxy for the costs of reaching zero-dose children—resulting in an average 2024 economic cost of $8.65 per dose and $112.45 per fully vaccinated zero-dose child across 129 LMICs.
- This study provides an evidence base to estimate the investments needed to close immunization coverage gaps under the Immunization Agenda 2030.

## Introduction

A significant public health concern in low- and middle-income country (LMIC) settings is the persistence of children who have not received any routine vaccinations—commonly referred to as ‘zero-dose’ children (World Health Organization 2020). The World Health Organization’s Immunization Agenda 2030 aims to fully vaccinate all people with immunization services, including ‘zero-dose’ children (World Health Organization). Despite decades of progress in improving immunization coverage, the number of children remaining unvaccinated has increased in recent years, leaving them highly susceptible to preventable diseases such as measles, polio, diphtheria, and pertussis (World Health Organization 2023). This gap in immunization not only contributes to elevated morbidity and mortality rates among unprotected children but also undermines broader efforts to achieve herd immunity and control outbreaks. The presence of zero-dose children is often concentrated in marginalized populations facing systemic barriers such as poverty, conflict, displacement, and weak health infrastructure (Wendt et al. 2022, Hogan and Gupta 2023).

We have limited direct evidence quantifying the costs of fully vaccinating zero-dose children with needed vaccinations and the evidence that does exist reveals important gaps. For example, Clarke-Deelder et al. (2024) examined India’s Intensified Mission Indradhanush, and estimated the incremental cost of the campaign at about US$82.99 per zero-dose child reached (with wide uncertainty) from the program perspective, and show that the strategy was likely cost-effective under per-capita GDP thresholds (Clarke-Deelder et al. 2024). Meanwhile, Portnoy et al. (2020) provided “standardized” delivery unit cost estimates across LMICs (excluding vaccine price) showing an average cost of US$1.87 per vaccine dose delivered via routine services, though this does not specifically isolate zero-dose children or special outreach efforts (Portnoy et al. 2020). Additionally, a commentary by Portnoy et al. (2021) highlights that relatively few immunization cost studies have estimated marginal or scale-up costs or examined how costs vary by coverage levels, reflecting the methodological challenges of measuring the additional investments needed to reach zero-dose populations (Portnoy et al. 2021). More recently, Levin et al. (2024) carried out a scoping review of interventions explicitly aiming to reach zero-dose children in LMICs, identifying eleven such studies; intervention costs ranged widely—from about US$0.08 per additional dose for low-touch strategies like SMS reminders in Kenya to about US$67 per dose for more intensive interventions such as cash transfers in Nicaragua (Levin et al. 2024).

In order to design and budget for targeted interventions to reduce disease burden and advance health equity globally, decision-makers need to know how much it will cost to fully vaccinate zero-dose children with needed vaccinations. The objective of this study was to approximate the costs of reaching zero-dose children by producing standardized country-level estimates of outreach delivery costs for all countries meeting the World Bank’s LMIC classification (World Bank 2025a). The identified costs were analyzed in a Bayesian meta-regression analysis framework based on the previously published Portnoy et al. (2020) analysis (Portnoy et al. 2020).

## Materials and methods

### Study data

We relied on a publicly available database describing immunization delivery costs in LMIC settings—the Immunization Delivery Cost Catalogue (IDCC) maintained by the Immunization Costing Action Network (ICAN) (Vaughan et al. 2019, Immunization Costing Action Network (ICAN) 2024). The IDCC is an online web catalog and downloadable Excel spreadsheet of immunization delivery cost evidence in LMIC settings, which describes the results of a systematic review of published and gray literature (covering three categories of key words: “immunization” AND “cost” AND “delivery”) available between January 2005 and December 2023. From the IDCC, we identified studies that reported delivery costs of outreach and mobile delivery efforts for childhood vaccines to fully vaccinate children up to the age of 15 with needed immunizations. “Outreach” was defined by IDCC as vaccines delivered through outreach or mobile clinics, whether as part of the routine immunization program or through a mass vaccination campaign. Delivery costs included labor, supply chain, capital, and other service delivery costs. ‘Supply chain’ includes costs for cold chain equipment, vehicles, transport, and fuel; “other service delivery” includes costs for program management (i.e. supervision and monitoring), training, social mobilization, and disease surveillance (Brenzel et al. 2015).

For the identified studies, we extracted the study year, estimates of the delivery cost per dose, whether the vaccine was delivered through routine outreach/mobile delivery vs. mass vaccination campaigns [Routine], whether the costing was full or incremental [Full], and whether the presented costs were from the financial or economic perspective [Econ]—i.e. financial costs refer to expenditures or financial outlays whereas economic costs include both financial costs as well as the opportunity cost associated with using inputs in the immunization program as compared to their next best use. Routine, Full, Econ are all binary indicator variables with possible values of 0 and 1 indicating the absence (0) or presence (1) of the corresponding characteristic. Where the IDCC did not include a cost per dose (excluding vaccine costs) directly reported by the study, we reviewed the original article and calculated the cost per dose from available data.

The IDCC 2024 update included delivery cost per dose estimates in 2022 US dollars (USD). In order to present estimates in 2024 US dollars, the study values were inflated using local inflation according to the consumer price index and local currency-to-USD exchange rates.

### Model specification

We compiled data on country-level covariates potentially associated (Portnoy et al. 2020) with outreach delivery unit costs: log gross domestic product (GDP) per capita [log(GDP)], log total under-five population [log(Pop)], diphtheria-tetanus-pertussis (DTP1) first dose coverage (ranging from 0 (0% coverage) to 1 (100%) coverage) [DTP1], log under-five mortality rate (under-five deaths per 1000 live births) [log(U5MR)], log population density (people per square kilometer of land) [log(Density)], and urban population (percentage of country population living in urban areas) [Urban] (United Nations 2024, World Bank 2025b, World Health Organization 2025).

Country-level covariates were compiled for the year of data collection for each costing study and combined with study-level indicators, i.e. Year,Routine, Full, Econ. By using a log transformation of specified covariates, we assumed these explanatory factors relate to the outcome on the multiplicative scale rather than linearly (e.g. a doubling in per-capita GDP produces a fixed increase in the logged outcome).

We used a Bayesian meta-regression model to regress outreach delivery unit costs against country-level and study-level explanatory variables. Continuous variables were standardized to a mean of zero and standard deviation of one before fitting the regression model. We constructed a prediction model for the intervention cost per dose outcome ($y_{i}$), specified as a generalized linear regression model, assuming a Gamma-distributed outcome and a log link function. In addition to study-level predictors, we selected country-level covariates according to the best fit model using minimized Watanabe-Akaike Information Criterion. The model specification assumed a Gamma likelihood function for the observed data $y_{i}$ where the shape parameter *α* described the residual variance:


(1) $$y_{i}\;∼\;\mathrm{Gamma}\left(\alpha ,\frac{\alpha }{c_{i}}\right)$$

This specification assumed variance proportional to $c_{i}$, representing the mean estimate of the delivery cost per dose for a given study. We assumed informative prior distributions for all regression coefficients, which were assumed to follow a normal distribution centered at zero with a standard deviation of 1 (Gelman 2025). The shape parameter ­was assumed to follow a half-Cauchy distribution centered at zero with a standard deviation of 5 (Gelman 2006).

The prediction model was estimated in R software, version 4.4.1, using an adaptive Hamiltonian Monte Carlo algorithm in the Stan software package, version 2.32.6, with four chains of 5000 iterations. The first 2500 iterations were discarded (burn-in period), yielding 10 000 posterior draws for analysis (Hoffman and Gelman 2014).

Stan model diagnostics (posterior summaries, convergence diagnostics, sampling details, and model fit assessment) were examined to determine any problems encountered by the sampler, and the potential scale reduction factor (i.e. R-hat) for all parameters was evaluated to confirm that the model had successfully converged. We simulated and visually inspected the ability of the model to reproduce the observed distribution of costs.

### Predicted outcomes

The fitted prediction model was used to generate economic outreach delivery cost per dose estimates for each country for the year 2024. As a proxy for the costs of fully vaccinating zero-dose children, we predicted delivery costs assuming routine outreach delivery and a full costing approach. To generate these estimates, we predicted values from the posterior distributions of the fitted model, with covariate values specific to each country and year. We generated aggregated estimates of the cost per dose in all LMICs and in country groups defined by World Bank income level (low-income, lower middle-income, and upper middle-income) by weighting country-specific estimates by the size of the under-five population in each country (World Bank 2025a). We tested predictive performance by comparing model predictions to the observed cost per dose matched to country and year.

### Sensitivity analysis

We conducted a sensitivity analysis excluding identified outliers to assess the robustness of model estimates to influential observations. Statistical outliers were defined as observations with cost per dose values greater than 1.5 times the interquartile range above the third quartile.

## Results

### Regression model

We identified 22 studies including 48 observations across 19 countries with outreach delivery costs for childhood vaccines reported in the IDCC. Among these studies, the outreach delivery cost per dose ranged from $0.09 to $9.80 in 2024 USD. Table 1 provides summary information on the empirical studies used in the analysis (full details of these studies are provided in Supplementary Appendix Table A).

**Table 1:** Summary characteristics for outreach delivery unit cost per dose data.

Variable	Estimates (*n*)
Income	
LI	24
LMI	22
UMI	2
Cost type	
Economic	27
Financial	21
Costing approach	
Full	21
Incremental	27
Delivery modality	
Routine	37
SIA	11

Note: LI, low-income; LMI, lower middle-income; SIA, supplementary immunization activities; UMI, upper middle-income.

In the sample, the average GDP per capita was $1743.57 (SD $1398.95), average population size was 16 700 (SD 32 900), and the average DTP1 coverage was 92.7% (SD 6.8%). The best fit model specification was defined as:


(2) $$\begin{array}{rl} c_{i} & =\exp(\beta_{0}+\beta_{1}*\mathrm{Yea}r_{i}+\beta_{2}*\mathrm{Eco}n_{i}+\beta_{3}*\mathrm{Ful}l_{i}+\beta_{4}*\mathrm{Routin}e_{i}+\\ & \;\beta_{5}*\log\;(\mathrm{GDP}{)}_{i}+\beta_{6}*\log\;(\mathrm{Pop}{)}_{i}+\beta_{7}*\mathrm{DTP}1_{i}) \end{array}$$

**Figure 1:**
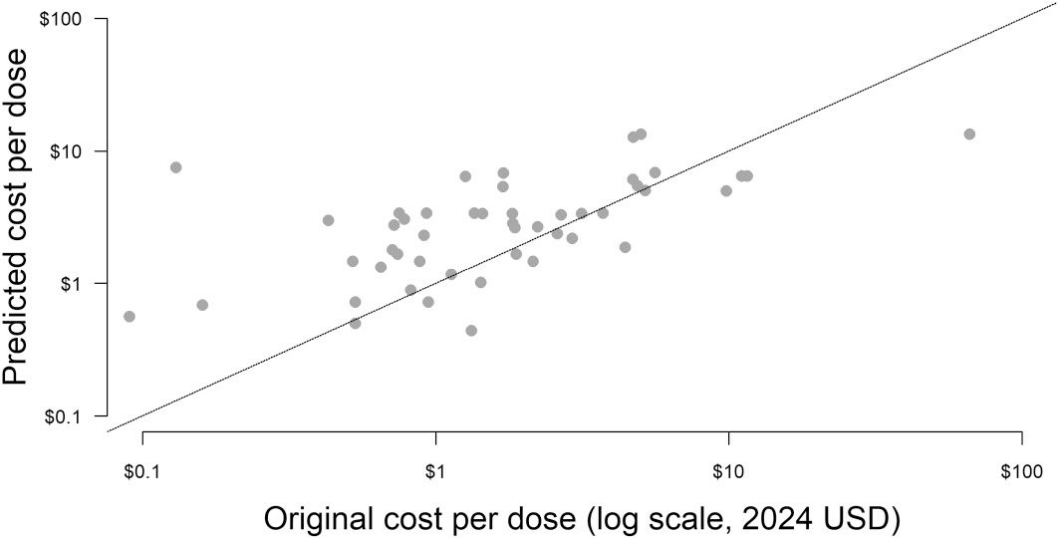
Comparison of predicted cost per dose and published literature cost per dose for outreach delivery.

Table 2 reports point estimates and standard errors for regression coefficients and other model parameters. The best fit model demonstrated satisfactory convergence diagnostics, with R-hat values below 1.01 for all estimated parameters and adequate effective sample sizes. Figure 1 displays the in-sample fit comparing observed versus predicted posterior values for the study sample.

**Table 2:** Results for regressions of outreach delivery unit cost per dose on predictors.

Variable	Mean coefficient (standard error)
Intercept	−0.158 (0.006)
Year	− 0.191 (0.003)
Economic cost indicator [Econ]	0.830 (0.003)
Full costing indicator [Full]	0.083 (0.005)
Routine delivery indicator [Routine]	0.727 (0.004)
log(GDP)	0.126 (0.002)
log(Pop)	0.475 (0.002)
DTP1 coverage	0.525 (0.001)
Gamma dispersion parameter alpha	1.412

**Figure 2:**
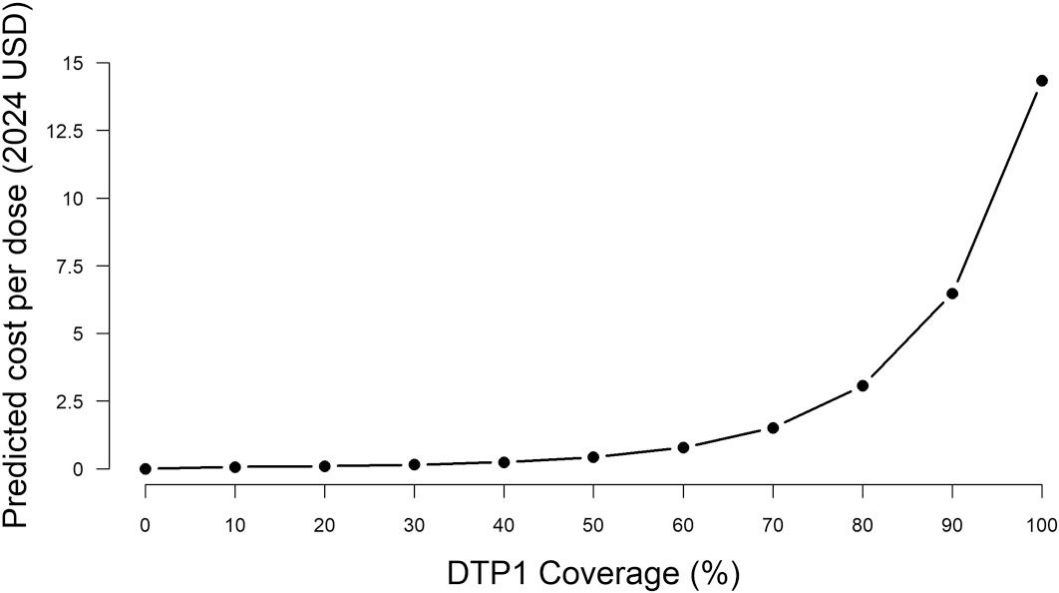
Predicted economic cost per dose in 2024 for routine outreach vaccine delivery by DTP1 (first dose diphtheria-tetanus-pertussis-containing vaccine) coverage for 129 low and middle-income countries.

The fitted model demonstrated increasing costs as price levels (GDP per capita), service delivery volume (under-five population), health system capacity (DTP1 coverage) increased. We calculated first differences to describe the percentage difference in the cost per dose associated with specified changes in individual predictors, holding others fixed. Doubling per-capita GDP would be associated with a 6.7% increase in the cost per dose of outreach-based delivery, whereas doubling the under-five population would be associated with a 34.4% increase. An increase in DTP1 coverage by one percentage point would be associated with a 7.7% increase in the cost per dose of outreach-based delivery. Figure 2 shows how the estimated outreach-based delivery cost per dose varies with coverage, holding all other covariates constant.

### Predicted outcomes

**Figure 3:**
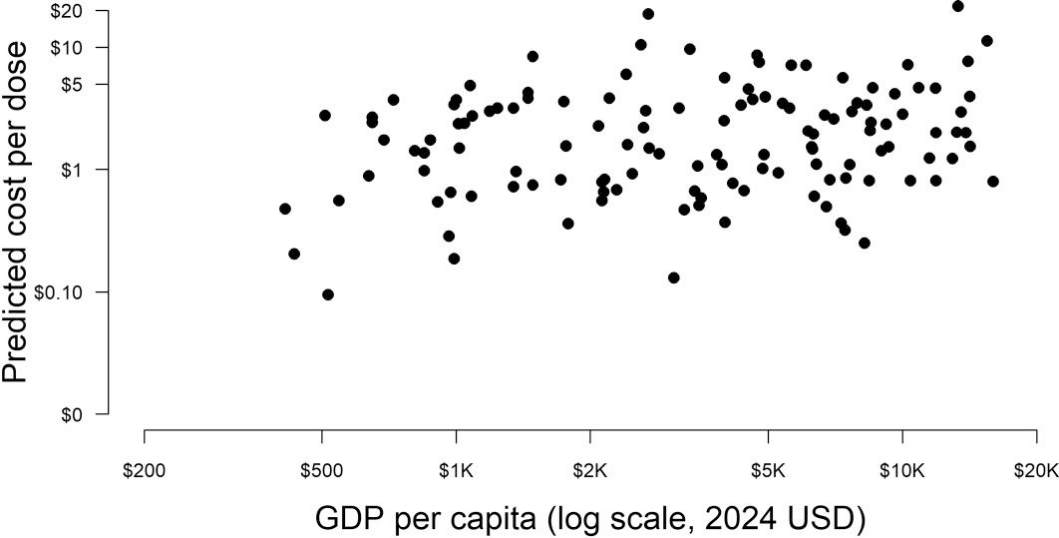
Predicted economic cost per dose in 2024 for routine outreach vaccine delivery by gross domestic product (GDP) per capita for 129 low and middle-income countries.

For the year 2024, the population-weighted average economic cost per dose was estimated to be $8.65 (95% credible interval $2.33–23.71) for the full costs of routine outreach vaccine delivery across 129 LMICs. By income level, the average predicted economic cost per dose was $2.09 ($0.59–5.47) for low-income countries, $9.91 ($2.59–27.48) for lower middle-income countries, and $10.88 ($2.37–33.93) for upper middle-income countries. Figure 3 presents the country-level cost per dose estimates by GDP per capita and 2025 World Bank income level. The set of country-specific economic cost estimates for outreach delivery cost per dose can be found in Table 3.

**Table 3:** Low- and middle-income country parameters with predicted economic outreach delivery cost per dose in 2024.

Country	World Bank income level^a^ (World Bank 2025a)	WHO region	Per-capita gross domestic product (World Bank 2025b)	Under-five population (thousands) (World Bank 2025b)	DTP1 coverage (World Health Organization 2025)	Predicted economic outreach delivery cost per dose for childhood vaccines
Afghanistan	LI	EMR	414	6656	0.66	$0.48
Albania	UMI	EUR	10 012	145	0.99	$2.84
Algeria	UMI	AFR	5631	4753	0.98	$7.14
Angola	LMI	AFR	2122	6219	0.7	$0.79
Argentina	UMI	AMR	13 858	2751	0.8	$2.00
Armenia	UMI	EUR	8501	177	0.97	$2.43
Azerbaijan	UMI	EUR	7284	675	0.65	$0.36
Bangladesh	LMI	SEAR	2593	16 505	0.99	$10.50
Belarus	UMI	EUR	8317	399	0.98	$3.39
Belize	UMI	AMR	8430	36	0.89	$0.81
Benin	LMI	AFR	1485	2176	0.76	$0.75
Bhutan	LMI	SEAR	3839	48	0.98	$1.33
Bolivia	LMI	AMR	4001	1251	0.65	$0.37
Bosnia and Herzegovina	UMI	EUR	8957	130	0.91	$1.42
Botswana	UMI	AFR	7695	294	0.98	$2.98
Brazil	UMI	AMR	10 280	13 361	0.91	$7.19
Burkina Faso	LI	AFR	987	3351	0.95	$3.40
Burundi	LI	AFR	154	2167	0.89	$1.48
Cabo Verde	UMI	AFR	5273	39	0.93	$0.94
Cambodia	LMI	WPR	2628	1816	0.9	$2.21
Cameroon	LMI	AFR	1762	4393	0.82	$1.55
Central African Republic	LI	AFR	516	941	0.52	$0.09
Chad	LI	AFR	1016	3547	0.84	$1.50
China	UMI	WPR	13 303	58 042	0.97	$21.71
Colombia	UMI	AMR	7914	3553	0.89	$3.52
Comoros	LMI	AFR	1784	114	0.8	$0.36
Congo	LMI	AFR	2482	886	0.82	$0.92
Congo DR	LI	AFR	647	19 276	0.82	$2.43
Cote d'Ivoire	LMI	AFR	2710	4594	0.8	$1.50
Cuba	UMI	AMR	9605	504	0.99	$4.16
Djibouti	LMI	EMR	3496	115	0.82	$0.51
Dominica	UMI	AMR	10 405	4	0.96	$0.81
Dominican Republic	UMI	AMR	10 876	1004	0.97	$4.67
Ecuador	UMI	AMR	6875	1404	0.74	$0.83
Egypt	LMI	EMR	3338	11 781	0.99	$9.66
El Salvador	UMI	AMR	5580	496	0.98	$3.19
Equatorial Guinea	UMI	AFR	6745	252	0.75	$0.50
Eritrea	LI	AFR	689	461	0.97	$1.74
Ethiopia	LI	AFR	1011	19 067	0.81	$1.10
Fiji	UMI	WPR	6288	83	0.95	$2.37
Gabon	UMI	AFR	8219	331	0.62	$1.47
Gambia	LI	AFR	909	384	0.82	$0.25
Georgia	UMI	EUR	9194	242	0.95	$0.54
Ghana	LMI	AFR	2406	4226	0.99	$2.36
Grenada	UMI	AMR	11 872	7	0.93	$6.04
Guatemala	UMI	AMR	6150	1879	0.86	$0.81
Guinea	LMI	AFR	1717	2195	0.77	$2.08
Guinea-Bissau	LI	AFR	963	297	0.74	$0.83
Haiti	LMI	AMR	2143	1236	0.76	$0.29
Honduras	LMI	AMR	3426	1138	0.75	$0.66
India	LMI	SEAR	2697	114 665	0.96	$0.67
Indonesia	UMI	SEAR	4925	22 349	0.83	$18.67
Iran	UMI	EMR	4771	6201	0.98	$3.94
Iraq	UMI	EMR	6074	5560	0.97	$7.56
Jamaica	UMI	AMR	7020	166	0.99	$7.13
Jordan	LMI	EMR	4618	1163	0.97	$2.60
Kazakhstan	UMI	EUR	14 005	2126	0.99	$3.75
Kenya	LMI	AFR	2206	7037	0.91	$7.70
Kiribati	LMI	WPR	2289	16	0.96	$3.84
Korea DPR	LI	SEAR	1447	1711	0.99	$0.69
Kyrgyz Republic	LMI	EUR	2419	788	0.9	$3.85
Lao PDR	LMI	WPR	2124	796	0.76	$1.60
Lebanon	LMI	EMR	3478	452	0.86	$0.56
Lesotho	LMI	AFR	972	266	0.86	$1.07
Liberia	LI	AFR	846	773	0.91	$0.65
Libya	UMI	EMR	6318	644	0.9	$1.37
North Macedonia	UMI	EUR	9310	95	0.93	$1.95
Madagascar	LI	AFR	545	4588	0.7	$0.56
Malawi	LI	AFR	508	3080	0.94	$2.78
Malaysia	UMI	WPR	11 867	2271	0.93	$4.65
Maldives	UMI	SEAR	13 216	31	0.99	$2.02
Mali	LI	AFR	1086	4158	0.91	$2.75
Marshall Islands	UMI	WPR	7467	4	0.98	$0.85
Mauritania	LMI	AFR	2083	800	0.95	$2.28
Mauritius	UMI	AFR	11 872	61	0.97	$2.00
Mexico	UMI	AMR	14 158	10 309	0.83	$3.97
Micronesia	LMI	WPR	4166	12	0.96	$0.77
Moldova	UMI	EUR	7618	179	0.87	$1.10
Mongolia	UMI	WPR	6691	353	0.97	$2.79
Montenegro	UMI	EUR	12 935	37	0.92	$1.23
Morocco	LMI	EMR	3993	3180	0.98	$5.62
Mozambique	LI	AFR	647	5669	0.9	$2.68
Myanmar	LMI	SEAR	1359	4432	0.76	$0.96
Namibia	LMI	AFR	4413	409	0.79	$0.67
Nepal	LMI	SEAR	1447	2792	0.98	$4.29
Nicaragua	LMI	AMR	2848	657	0.88	$1.34
Niger	LI	AFR	723	4667	0.95	$3.72
Nigeria	LMI	AFR	807	33 527	0.71	$1.43
Pakistan	LMI	EMR	1485	31 789	0.94	$1.53
Papua New Guinea	LMI	WPR	3076	1229	0.49	$8.40
Paraguay	UMI	AMR	6416	679	0.82	$0.13
Peru	UMI	AMR	8452	2692	0.83	$1.11
Philippines	LMI	WPR	3985	9602	0.82	$2.09
Rwanda	LI	AFR	1000	1878	0.99	$2.51
Samoa	UMI	WPR	4899	28	0.99	$3.74
Sao Tome and Principe	LMI	AFR	3245	31	0.87	$1.33
Senegal	LMI	AFR	1744	2452	0.96	$0.47
Serbia	UMI	EUR	13 524	315	0.95	$3.60
Sierra Leone	LI	AFR	873	1164	0.92	$2.97
Solomon Islands	LMI	WPR	2149	104	0.91	$1.74
Somalia	LI	EMR	637	3412	0.78	$0.83
South Africa	UMI	AFR	6253	5888	0.76	$0.89
South Sudan	LI	AFR	1080	1484	0.76	$1.53
Sri Lanka	LMI	SEAR	4516	1635	0.98	$0.61
St. Lucia	UMI	AMR	14 182	10	0.99	$4.55
St. Vincent and the Grenadines	UMI	AMR	11 501	6	0.99	$1.54
Sudan	LI	EMR	989	7646	0.48	$1.24
Suriname	UMI	AMR	7431	53	0.75	$0.19
Eswatini	LMI	AFR	3936	142	0.91	$0.32
Syrian Arab Republic	LI	EMR	847	2273	0.81	$0.98
Tajikistan	LMI	EUR	1341	1334	0.98	$3.18
Tanzania	LMI	AFR	1186	10 810	0.87	$3.01
Thailand	UMI	SEAR	7345	3102	0.96	$5.62
Timor-Leste	LMI	SEAR	1343	157	0.89	$0.73
Togo	LI	AFR	1043	1342	0.95	$2.40
Tonga	UMI	WPR	4864	12	0.99	$1.02
Tunisia	LMI	EMR	4350	902	0.97	$3.37
Turkiye	UMI	EUR	15 473	5746	0.99	$11.26
Turkmenistan	UMI	EUR	8572	804	0.99	$4.68
Tuvalu	UMI	WPR	6345	1	0.99	$0.60
Uganda	LI	AFR	1073	7938	0.95	$4.88
Ukraine	UMI	EUR	5389	1304	0.95	$3.49
Uzbekistan	LMI	EUR	3162	4311	0.9	$3.19
Vanuatu	LMI	WPR	3543	44	0.88	$0.58
Venezuela	UMI	AMR	15 944	2124	0.67	$0.80
Viet Nam	LMI	WPR	4717	7178	0.99	$8.63
Yemen	LI	EMR	433	6294	0.53	$0.21
Zambia	LMI	AFR	1235	3152	0.94	$3.18
Zimbabwe	LMI	AFR	2656	2307	0.93	$3.05

AFR, African region; AMR, Region of the Americas; DTP1 = diphtheria-tetanus-pertussis first dose coverage; EMR, Eastern Mediterranean region; EUR, European region; LI = low-income; LMI, lower middle-income; SEAR, Southeast Asian region; UMI, upper middle-income; WPR, Western Pacific region.

^a^LI: Gross national income (GNI) per capita of $1135 or less in 2024; LMI: GNI per capita $1136 and $44 915; UMI: GNI per capita of $4496 and $13 935.

### Sensitivity analyses

After excluding statistical outliers, the model fit showed certain meaningful changes in coefficient estimates, suggesting that a small number of extreme high-cost observations substantially influenced the original regression estimates (Supplementary Appendix Table B). The Gamma dispersion parameter alpha increased from 1.412 in the full dataset model to 2.272 after excluding outliers, indicating improved model fit and reduced influence of extreme observations on the residual distribution. This sensitivity analysis resulted in an estimated population-weighted average economic cost per dose of $6.12 ($2.23–13.92) for the full costs of routine outreach vaccine delivery across 129 LMICs, which overlaps with the associated estimates from the primary analysis including outliers, $8.65 ($2.33–23.71). Country-specific economic cost estimates for outreach delivery cost per dose based on the model excluding statistical outliers can be found in Supplementary Appendix Table C.

## Discussion

For the year 2024, the average economic cost per dose across all LMICs was estimated to be $8.65 ($2.33–23.71) for the full costs of routine outreach vaccine delivery, excluding vaccine costs. These estimates are consistent with the empirical estimates reported in a recent scoping review for the costs of interventions to reach zero-dose children, which ranged from $0.04 to $67 per dose (Levin et al. 2024, UNICEF 2024), but are notably greater than estimates of $2.34 ($0.80–5.47) in 2024 USD to deliver routine childhood vaccinations on average (Portnoy et al. 2020). Importantly, the estimates presented here should be interpreted as an approximation of the additional delivery resources potentially required to reach underserved populations through outreach and mobile strategies, rather than as direct estimates of the costs of reaching zero-dose children. These estimates can be useful in providing a broad indication of delivery costs to inform resource allocation to reach currently un- and under-reached populations with needed vaccinations.

Using these predicted routine outreach delivery costs per dose as a proxy for reaching zero-dose children, the costs of fully vaccinating a zero-dose child would be scaled by the number of vaccine doses in the vaccine program. For example, if we assume the vaccine program requires 13 doses (e.g. 3 pentavalent vaccine, 3 oral polio vaccine, 3 pneumococcal conjugate vaccine, 3 rotavirus, 1 measles-containing vaccine) for a child to be considered fully vaccinated, and we assume that zero-dose children can be reached at the average unit cost per dose of outreach delivery, the predicted average economic cost per dose would be multiplied by 13, which would be equivalent to $112.45 ($30.29–308.23) to fully vaccinate a zero-dose child across the study countries. However, this calculation should be interpreted as a simplified heuristic intended to illustrate the potential magnitude of delivery costs rather than as a realistic child-level costing model. In practice, the resources required to fully vaccinate a zero-dose child would depend on the child’s age, opportunities for co-administration of vaccines, visit schedules, and dropout between visits.

An alternative illustrative approach would take into account the observed positive association between coverage and unit costs, and therefore focus on the marginal cost per dose. Under this approach, for a country at 90% DTP1 coverage, the estimated marginal cost per dose of outreach-based delivery is $12.11; multiplied by 13, this would imply a cost of $157.43 to fully vaccinate an unvaccinated child under the same simplifying assumptions.

In the best fit model, the continuous explanatory variables included were GDP per capita, under-five population size, and DTP1 coverage. In our analysis, unit costs were predicted to increase with higher GDP per capita, larger under-five populations, and higher DTP1 coverage. The positive association with GDP per capita may reflect higher input and labor costs in higher-income settings, while the positive associations with under-five population and DTP1 coverage may reflect greater resource requirements associated with larger target populations and more extensive outreach efforts in stronger immunization systems. Finally, we assigned the year of data collection as one of the study-level covariates, which serves as a proxy for time-varying characteristics not captured by other model covariates. Costs were predicted to decrease over time, but the magnitude was small. However, it is important to note that regression coefficients from this model do not have a causal interpretation. Our sensitivity analysis results suggest that the primary substantive conclusions of the model were generally preserved, but that several covariate relationships were sensitive to a small number of influential high-cost observations.

There are several limitations to this analysis. First, the studies we included in the analysis did not have a standardized design, and the resulting heterogeneity was not fully captured by our meta-regression analysis. Therefore, the residual variance could reflect both sampling uncertainty as well as non-sampling error due to methodological heterogeneity. However, given the limited number of available studies, restricting the analysis to only narrowly comparable study designs would have substantially reduced the geographic scope and empirical basis of the analysis. We therefore combined evidence across heterogeneous study types to provide a broader evidence base across LMICs, while attempting to account for key methodological differences through study-level covariates in the meta-regression. The 19 countries included in the dataset might not be representative of the 129 LMICs to which we extrapolated predicted costs. Therefore, estimates will be less reliable for countries with covariate values outside the range observed in the analyzed sample—for example, countries with substantially higher or lower GDP per capita, under-five population size, or DTP1 coverage than those represented in the underlying studies. In the sample used to fit the model, the average GDP per capita was $1743.57 (std $1398.95), the average under-five population size was 16 700 (std 32 900), and the average DTP1 coverage was 92.7% (std 6.8%). Predictions for countries with covariate values broadly within these observed ranges are therefore more reflective of interpolation within the empirical data used for model fitting, whereas predictions for countries with values substantially outside these ranges rely more heavily on extrapolation beyond the observed data and should be interpreted with greater caution. In particular, only two observations from currently-defined upper middle-income countries were included in the dataset used for model fitting. Second, this analysis assumed that the estimated unit costs of vaccine delivery are independent of the number of doses in the immunization program. However, we would expect different costs for vaccines requiring unique immunization visits compared to vaccines that can be administered in the same immunization visit. Finally and importantly, outreach and mobile efforts to reach children already reached by ongoing immunization efforts might not be representative of efforts required to fully vaccinate zero-dose children, who are not reached by the programs informing this analysis by definition. While outreach and mobile delivery strategies are likely to play an important role in efforts to reduce the number of zero-dose children, the costs of outreach delivery should not be considered equivalent to the full costs of identifying and reaching zero-dose populations. We expect that fully vaccinating zero-dose children may be even more costly, not only because they are among the most difficult populations to reach, but also because identifying and locating zero-dose children can itself require substantial additional resources. Zero-dose children are often geographically dispersed, mobile, marginalized, or outside formal health system contact, making them challenging to identify through routine service delivery mechanisms. Future studies that focus on the costs of reaching zero-dose children should include the costs of identifying and locating those children to ensure that cost estimates reflect the full resource burden required. In particular, we expect higher costs in geographically hard-to-reach or conflict-affected settings, which likewise increase the risk of a child being zero-dose. Additional activities beyond vaccine delivery itself—including community engagement, population mapping, surveillance, and identification of previously unreached children—may contribute importantly to the total resources required to reduce zero-dose prevalence. Our cross-sectional analysis suggests that costs increase as coverage increases; if this relationship holds true longitudinally as individual countries increase coverage, then the average cost per dose would increase significantly as countries reach more children.

In light of these limitations, this analysis may not fully address the decision- and policy-making needs to improve immunization coverage and equity, and reach the Immunization Agenda 2030 goals. However, the need to make policy choices based on imperfect information is unavoidable, and the estimates we report provide an additional evidence source for budgeting and planning around closing immunization coverage gaps.

Our study provides estimates produced via meta-regression analyses that can help countries to improve budgeting and planning for implementation of future interventions to reach zero-dose children, offering critical insights for global health stakeholders and policymakers. These estimates are best interpreted as approximate indicators of the costs associated with outreach- and mobile-based delivery approaches that may contribute to improving coverage among underserved populations, including zero-dose children. Bridging the immunization gap for zero-dose children through routine systems not only advances universal health coverage but also strengthens health systems and improves population resilience to future public health threats (Oyugi et al. 2025). As countries and partners mobilize resources to close this immunization gap, these cost estimates serve as a valuable foundation for strategic planning, prioritization, and sustainable financing.

## HIC authorship

This study is a multi-country modeling analysis using secondary data extracted from published literature and available open access in an online database. Although the estimates are produced for research focused on low- and middle-income countries, the country-level analytic framework does not rely on local contextual factors. The authorship list includes HIC authors only.

## Supplementary Material

czag085_Supplementary_Data

## Data Availability

The data that support the findings of this study are openly available in the Immunization Delivery Cost Catalogue (IDCC) at https://immunizationeconomics.org/ican-idcc/. All data produced in the present work are contained in the manuscript and Supplementary Materials.

## Reflexivity statement

The authors include four females and one male and span multiple levels of seniority. While two of the authors are affiliated with academic institutions, three are affiliated with an independent public-private partnership and multilateral funding agency. All five authors have extensive experience conducting costing and economic analyses of vaccination programs.
